# Direct Effects of Polyploidization on Floral Scent

**DOI:** 10.1007/s10886-025-01641-y

**Published:** 2025-09-10

**Authors:** Elisabeth Schlager, Stefan Dötterl, John N. Thompson, Magne Friberg, Karin Gross

**Affiliations:** 1 Department of Environment and Biodiversity, University of Salzburg, Hellbrunner Strasse 34, Salzburg, 5020 Austria; 2 Department of Ecology and Evolutionary Biology, University of California, Santa Cruz, Santa Cruz, CA 95060 USA; 3 Department of Biology, Lund University, Kontaktvägen 13, Lund, SE-223 62 Sweden

**Keywords:** Polyploidy, Synthetic polyploidization, Neopolyploids, Established polyploids, Floral scent, Floral evolution

## Abstract

Polyploidy is an important driver of the evolution and diversification of flowering plants. Several studies have shown that established polyploids differ from diploids in floral morphological traits and that polyploidization directly affects these traits. However, for floral scent, which is key to many plant-pollinator interactions, only a few studies have quantified differences between established cytotypes, and the direct effects of polyploidization on floral scent are not yet known. We compared the floral scent of established polyploids and diploids from a natural mixed-ploidy population of the plant *Lithophragma bolanderi* (Saxifragaceae), a species pollinated by two highly specialized moth pollinators of the genus *Greya* (Prodoxidae). We also compared the floral scent of neopolyploids synthetically generated from diploids with the floral scent of the diploid progenitors to quantify the direct effects of polyploidization on floral scent. Established tetraploids had a higher floral scent emission rate, produced more scent compounds, and emitted a relative scent composition that differed from diploids. Neotetraploids differed in the same direction from diploids as established tetraploids from diploids, but to a lesser extent. Together, our results provide novel insights into the ways in which polyploidization reshapes floral scent, thereby potentially altering interactions between plants and pollinators.

**Supplementary Information:**

The online version contains supplementary material available at 10.1007/s10886-025-01641-y.

## Introduction

Polyploidy is an important driver of evolution and diversification of flowering plants (e.g., Soltis et al. 2009). Typically, polyploidization generates immediate postzygotic reproductive isolation from the diploid progenitors and may immediately induce phenotypic changes in floral morphological traits involved in plant-pollinator interactions (e.g., Clo and Kolář 2021; Gross et al. 2025), which may affect also prezygotic isolation through assortative pollinator visitation. Thus, pollinators can impose divergent selection on different cytotypes, which may reinforce divergence between polyploids and their diploid progenitors (Nuismer and Cunningham 2005). However, studies on immediate effects of polyploidization on floral scent, which plays a key role in pollinator attraction (Dötterl and Gershenzon 2023), are lacking.

We identified *Lithophragma bolanderi* (Saxifragaceae) as an ideal system to explore effects of polyploidization on floral scent. This self-incompatible, perennial herb is endemic to the western slopes of the Sierra Nevada, California, USA. Like several other *Lithophragma* species, *L. bolanderi* is involved in a coevolutionary interaction with the two highly specialized *Greya* (Prodoxidae) moth pollinators *G. politella* and *G. obscura*, with the former also being a seed parasite (Thompson 2005). *Lithophragma bolanderi* is highly variable both in floral morphology (Thompson et al. 2017) and scent (Friberg et al. 2019), and its floral scent is an important attractant for female *G. politella* moths (Friberg et al. 2014). In addition, *L. bolanderi* comprises three major– diploid, tetraploid, and hexaploid– and three minor– triploid, pentaploid, and octoploid– cytotypes with one to four cytotypes per population (Gross et al. 2025). Polyploids of *L. bolanderi* most likely arose from multiple autopolyploidization events with potential introgression into some polyploid lineages from the congener *L. glabrum* (Gross et al. 2025). Plant ploidy explains some of the local variation in floral morphology in *L. bolanderi*, and, whereas polyploidization directly induced changes in floral size, selection mediated by the *Greya* moths has been suggested to affect local divergence in floral shape (Thompson et al. 2017; Gross et al. 2025). It is not yet known how polyploidy affects floral scent in *L. bolanderi*.

Here, we assess differences in floral scent between established diploids and tetraploids from a natural *L. bolanderi* population and the direct effects of polyploidization on floral scent in *L. bolanderi*. We experimentally generated neopolyploids using a colchicine treatment and compared the floral scent of these neopolyploids to the floral scent of established diploids and tetraploids as well as to plants treated with colchicine but remaining diploid. Our study contributes to the understanding of the significance of polyploidization on floral scent diversification and the interaction with pollinators.

## Methods and Materials

### Induction of Polyploidization and Plant Growing

The plants used in this study were also used in Gross et al. (2025) to quantify the direct effects of polyploidization on floral morphology in *L. bolanderi.* All individuals derived from seeds collected in a natural population in the canyon of the North Fork of the Kaweah River in California, USA (KAW; 36.484° N, −118.920° E, 345 m a.s.l.) in which diploids and tetraploids grow sympatrically. Polyploidization was induced by treating diploid seedlings with a 0.2% colchicine solution. Colchicine-treated plants were cross-pollinated to generate F1 plants to minimize side effects of the colchicine treatment. Diploid and tetraploid plants treated only with deionized H_2_O were used as control plants. The ploidy level of all F1 plants of the colchicine-treated group and of a subset of the F1 plants of the diploid and the tetraploid control groups was verified by flow cytometry at Plant Cytometry Service (https://www.plantcytometry.nl/). A total of 84 plants were available (*n*_diploid control plants_ = 22; *n*_tetraploid control plants_ = 22; *n*_colchicine−treated plants that remained diploid_ = 17; *n*_neotriploids_ = 16; *n*_neotetraploids_ = 7) resulting from 46 crosses between plants from 15 seed families (i.e. collected from different plant individuals in the field). Neotriploids were most likely the result of crosses involving at least one ploidy chimeric parental plant. Details of plant growth conditions and the generation of synthetic neopolyploids are described in Text S1 and S2, respectively.

### Scent Collection and Analysis

When F1 plants were flowering, floral scent was collected from 9.7 ± 3.8 (mean ± 1 standard deviation) flowers per plant using headspace sorption in a laboratory (20–21 °C; artificial and natural light) between 9:00 and 18:00 when *L. bolanderi* flowers are most scented (Friberg et al. 2014). Air from the headspace was sucked through a scent trap containing a 1:1 mixture of Tenax-TA (mesh 60–80; Supleco, Germany) and Carbotrap B (mesh 20–40; Suplec, Germany) for 15 min at a constant flow rate of 200 ml min^−1^ (for details, see Text S3). As controls, ambient air (empty oven bag) and leaves or a stalk with buds were sampled at each sampling occasion to control for background/green leaf volatile compounds.

The samples were analyzed using thermal desorption-gas chromatography/mass spectrometry (TD-GC/MS) on a ZB-5 fused silica column (Zebron™, Phenomenex Inc., USA) and with helium as carrier gas (for details, see Text S3). Chromatograms were processed using the GCMSolution package, version 4.50 (Shimadzu, Tokyo, Japan). The components were identified by comparing mass spectra and linear retention indices (RIs) with those in the data bases NIST11, Wiley9, Essential oils, FFNSC 2, and Adams (2007) and verified, when possible, with authentic reference compounds available in the Plant Ecology Lab at the University of Salzburg. For a (semi)automatic detection and integration of the floral scent compounds, a custom library was compiled based on the floral scent compounds previously detected in *L. bolanderi* (Friberg et al. 2019) and compounds additionally detected by screening our samples. Only compounds with a peak twice as large as the largest control sample peak and that were present in at least four samples were considered as floral scent compounds.

Peak areas of floral sent compounds were converted to absolute amounts in ng h^−1^ per flower based on an external standard including benzaldehyde, (*Z*)−3-hexenyl acetate, and linalool (racemic mixture) to calculate the emission rate. The relative amount of compounds was calculated by dividing the absolute amount of each compound by the total absolute amount of the sample.

### Statistical Analyses

We tested for differences between established diploids and tetraploids and for direct effects of polyploidization on floral scent, that is, whether neopolyploids differed from diploids, running a permutational multivariate analysis of variance (PERMANOVA) each for scent emission rate, for the number of scent compounds, and for relative scent composition (relative amounts) in PRIMER 6.1.15 with PERMANOVA + for PRIMER 1.0.5. All data were fourth-root-transformed, and Euclidean distances were computed for the scent emission rate and the number of compounds and Bray-Curtis similarities for relative amounts. Plant ploidy, colchicine treatment, and the ploidy-treatment interaction were included as fixed factors and the cross-specific combination of the parental seed families nested within the ploidy of the founder plants as random factor. Pairwise *posthoc* comparisons among the five groups were computed with PERMANOVAs with a single fixed factor ploidy-treatment group. For the pairwise comparison between colchicine-treated plants that remained diploid and neotetraploids, the random factor cross-specific combination of the parental seed families had to be nested within the ploidy-treatment group because nesting within the ploidy of the founder plants was not possible for the individuals available. We ran a permutational analysis of multivariate dispersion (PERMDISP) in PRIMER 6.1.15 with PERMANOVA + for PRIMER 1.0.5 for each of the three scent measures with the ploidy-treatment group as factor to test for differences in dispersion among groups. Differences in scent composition were visualized by performing non-metric multidimensional scaling (NMDS) on Bray-Curtis similarities of fourth-root-transformed relative amounts using the package *vegan* (v.2.6-4) in R 4.0.3.

We used the data for a specific crossing lineage that comprised three established diploids, three neotriploids, and three neotetraploids, and for another crossing lineage that comprised two established diploids and two colchicine-treated plants that remained diploid to assess effects of polyploidization independent of seed family effects using PERMANOVAs and PERMDISPs.

All *P*-values were computed with the number of permutations set to 9999. Type II sum of squares were used for models without interactions in the fixed effects and type III sum of squares for models with interactions. The proportion of the total variance explained was calculated by dividing the sum of square of the factor by the total sum of squares. *Posthoc* PERMANOVAs provide uncorrected *P*-values, which we use. Additionally, however, we computed Benjamini-Hochberg corrected *P*-values using the package *FSA* (v0.8.32) in R 4.0.3 (see Table S3).

## Results

Across samples, we detected 42 floral scent compounds (Table S1). This is less than the 71 compounds found by Friberg et al. (2019), and only 27 compounds were detected in both studies. This could partly be due to different scent collection and analysis methods but is most likely a result of the high among population variation in *L. bolanderi* (Friberg et al. 2019), and that our population was not among the 18 populations sampled by Friberg et al. (2019). Five compounds– linalool, methyl benzoate, methyl salicylate, 2-aminobenzaldehyde, and methyl anthranilate– were present in all 84 samples, and 14 compounds were detected in at least 50% of the samples (Table S1). Four compounds– 1,4-dimethoxybenzene, the two phenylacetaldoxime isomers, and phenylacetaldehyde– were only detected in the two established cytotypes (Table S1). Three compounds– 2-phenylethanol, 4-methoxybenzenemethanol, and unknown 6– were only detected in established tetraploids, whereas three compounds– (*E*)-β-ocimene, hotrienol, and unknown 2– were only detected in at least some samples of established diploids and of the three colchicine-treated cytotypes (Table S1). (*E*)-Cinnamyl alcohol was detected in established tetraploids and in at least some samples of the three colchicine-treated cytotypes and unknown 1 was detected in established tetraploids and in one neotetraploid but not in any of the other treatment groups (Table S2).

**Fig. 1:**
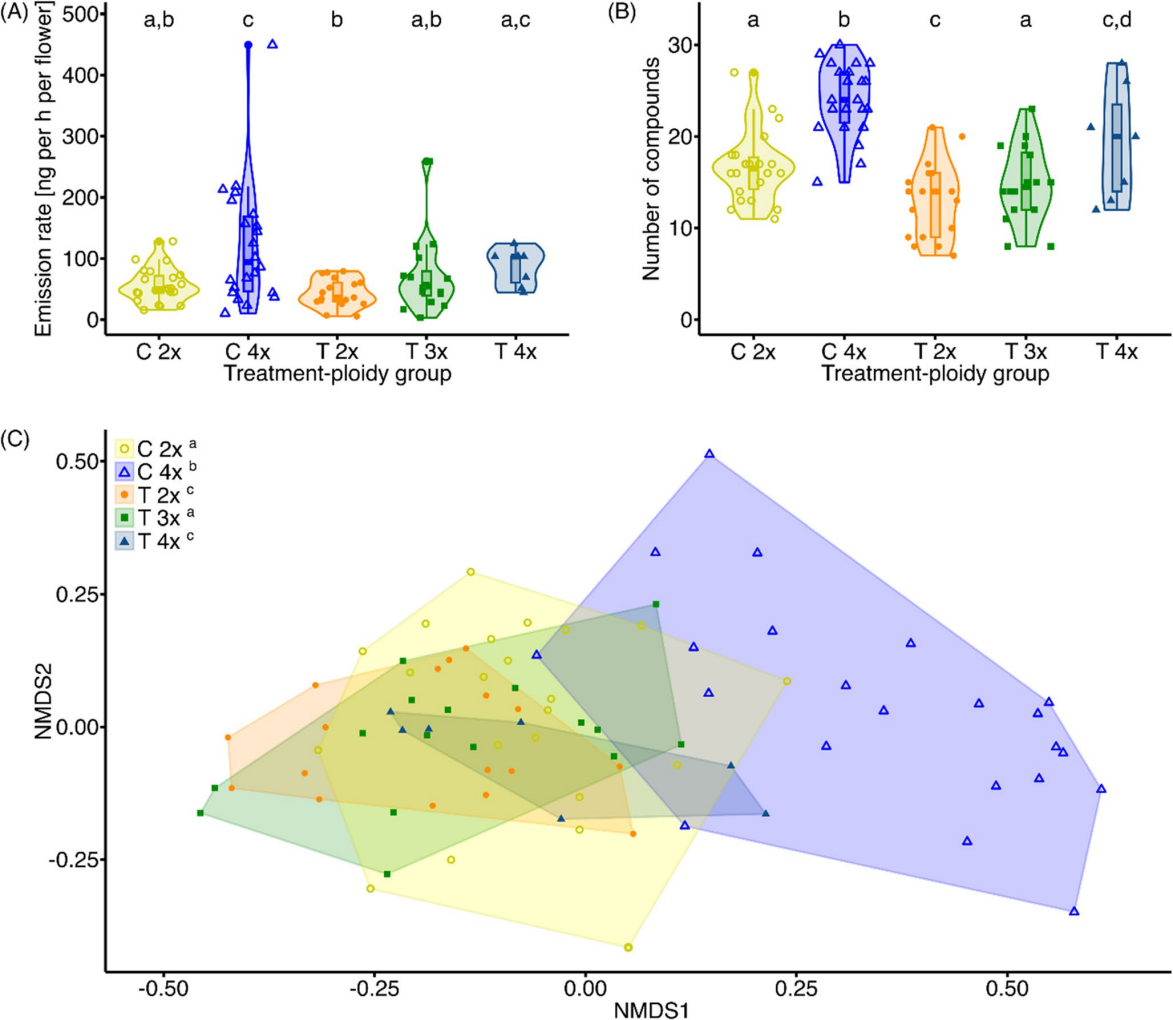
Floral scent of established diploids (“C 2x””) and tetraploids (“C 4x”) as well as of neopolyploids generated through colchicine treatment of diploid seedlings (“T 3x”: neotriploids, and “T 4x”: neotetraploids) and colchicine-treated plants that remained diploid (“T 2x”) of *Lithophragma bolanderi*. Differences among these five groups in the scent emission rate (**A**), the number of scent compounds (**B**), and in the relative amounts of compound (**C**). Points representing individuals, violin plots, boxplots, and areas are color-coded according to treatment and cytotype (see legend in (**C**)). Boxplots in (**A**) and (**B**) indicate the median, the first and third quartile, maximum and minimum values. Different lowercase letters at the top of the graph in (**A**) and (**B**)) and to the right of the legend in (**C**) indicate significant (*P* < 0.05; uncorrected *P*-values; for adjusted *P*-values, see Table S3) differences in pairwise *posthoc* comparisons (PERMANOVAs) among the five groups

Polyploids (established and experimentally induced combined) had a higher scent emission rate (Fig. 1A), more floral scent compounds (Fig. 1B), and a different relative scent composition (Fig. 1C; Fig. S1; Table S2) than diploids (Table 1). Relative amounts of compounds also differed between colchicine-treated plant and established cytotypes (Fig. 1C; Table 1). The cytotype-treatment interaction was not significant for any of the three scent measures (Fig. 1; Table 1). Plant ploidy level explained a higher proportion (4.5–12.8%) of the total variation than either colchicine treatment (0.9–3.3%) or the ploidy-treatment interaction (0.02–1.2%) for all three scent measures (Table 1).

Pairwise comparisons showed, when comparing the two established cytotypes, that established tetraploids had a higher scent emission rate and more scent compounds (Fig. 1A and B, Table S3). Moreover, established tetraploids differed in relative scent composition from established diploids (Fig. 1C, Table S3).

Comparisons between neotetraploids and diploids showed that neotetraploids had a higher scent emission rate than colchicine-treated plants that remained diploid (this effect was no longer significant when the *P*-value was adjusted) (Fig. 1A, Table S3) and more scent compounds than established diploids (Fig. 1B, Table S3). Neotetraploids had also a different relative scent composition than established diploids (Fig. 1C, Table S3).

When comparing neotetraploids with established tetraploids, there was no significant difference in the scent emission rate, but neotetraploids emitted fewer compounds (no longer significant when the *P*-value was adjusted) and had a different relative scent composition (Fig. 1, Table S3).

When comparing colchicine-treated plants that remained diploid to established diploids, there was no significant difference in the scent emission rate, but colchicine-treated plants that remained diploid emitted fewer compounds and at different relative amounts (no longer significant when the *P*-value was adjusted) (Fig. 1, Table S3).

Dispersion (variance) differed among the two established and the three colchicine-treated cytotypes only for the scent emission rate but not for the number of compounds and relative scent amounts, and for all scent measures most pairwise comparisons were non-significant (Fig. 1; Table S4).

When focusing on crosses for which we had at least two individuals from at least two out of our five established and colchicine-treated cytotypes, we found similar, albeit mostly non-significant, trends as across all individuals (Figs. S2 and S3; Table S5). Plant ploidy level explained between 28.6% and 61.2% of the total variation depending on the floral scent measure, and the colchicine treatment between 57.9% and 94.4% (Table S5).

**Table 1:** Statistical output of permutational multivariate analyses of variance (PERMANOVAs) for the scent emission rate, the number of scent compounds, and the relative amount of compounds

Trait	Factor	df	SS	MS	Pseudo-F	*P*	No. unique permutations	*R*^2^ [%]
Scent emission rate								
	Ploidy	2	1.52	0.76	3.22	**0.048**	9958	5.9
	Treatment	1	0.73	0.73	3.06	0.086	9815	2.9
	Ploidy × Treatment	1	0.31	0.31	1.31	0.263	9854	1.2
	Parental seed family (Original ploidy)	30	9.39	0.31	1.34	0.187	9908	36.6
	Residuals	49	11.42	0.23				44.6
	Total	83	25.62					
	Marginal effects							8.8
Number of compounds								
	Ploidy	2	0.3228	0.1614	12.59	**< 0.001**	9949	12.8
	Treatment	1	0.0225	0.0225	1.68	0.205	9837	0.89
	Ploidy × Treatment	1	0.0004	0.0004	0.03	0.858	9831	0.02
	Parental seed family (Original ploidy)	30	0.7218	0.0241	1.94	**0.020**	9906	28.6
	Residuals	49	0.6092	0.0124				24.2
	Total	83	2.5217					
	Marginal effects							33.5
Relative amounts								
	Ploidy	2	2064.9	1032.5	3.59	**0.001**	9917	4.5
	Treatment	1	1495.5	1495.5	4.96	**< 0.001**	9939	3.3
	Ploidy × Treatment	1	474.3	474.3	1.63	0.124	9934	1.0
	Parental seed family (Original ploidy)	30	17260.0	575.4	2.07	**< 0.001**	9801	38.0
	Residuals	49	13614.0	277.8				30. 0
	Total	83	45444.0					
	Marginal effects							23.2

Cytotype (Ploidy), treatment (Treatment), and the cytotype-treatment interaction (Ploidy × Treatment) were fixed factors, and the cross-specific combination of the parental seed families nested within the cytotype of the founder plants [Parental seed family (Original ploidy)] was a random factor. The proportion of the total variance explained (R^2^ [%]) is also indicated. Significant *P*-values are highlighted in bold

## Discussion

Only very few previous studies have assessed differences in floral scent between cytotypes (e.g., Gross and Schiestl 2015; Palmqvist et al. 2021). Our findings that established tetraploids had a higher scent emission rate, contained more floral scent compounds, and had a different relative scent composition than diploids from a natural mixed-ploidy population of *L. bolanderi* are consistent with previous studies in the orchid *Gymnadenia conopsea* (e.g., Gross and Schiestl 2015) and in *Chamerion angustifolium* (Onagraceae) (Palmqvist et al. 2021). Together, this indicates that differences in floral scent between established polyploids and diploids might be common and widespread, as it is the case for floral morphological traits (e.g., Gross and Schiestl 2015; Clo and Kolář 2021; Palmqvist et al. 2021; Gross et al. 2025).

Trait differentiation between established cytotypes may be a combined result of post-polyploidization differentiation through divergent selection or genetic drift, or a direct effect induced by the polyploidization process. Our experimental induction of a limited number of neopolyploids from diploids in one mixed-ploidy population of *L. bolanderi* suggests that polyploidization induces immediate qualitative and quantitative changes in floral scent. Our results also indicate that some of the floral scent variation may be attributed to the colchicine treatment and future studies using a higher sample size might, for example, try to better quantify and discriminate between colchicine-induced (potentially mediated by changes in DNA methylation or gene expression) and polyploidization-induced changes in floral scent. Successful neopolyploids, however, differed from colchicine-treated plants that remained diploid in the same direction as established tetraploids differed from established diploids suggesting that polyploidization affects scent differently than colchicine-induced effects. Thus, polyploidization can immediately induce phenotypic changes not only for morphological traits (cf. e.g., Clo and Kolář 2021; Gross et al. 2025) but also for olfactory floral traits.

For macroscopic morphological traits, a recent meta-analysis has shown that polyploids are larger than diploids and that polyploidization immediately induced these differences, because these traits did not differ between neopolyploids and established polyploids (Clo and Kolář 2021). A recent study in *L. bolanderi* (Gross et al. 2025) supports the observation that polyploidization immediately induces mainly an increase in flower size but less a change in floral shape and indicates that neopolyploids can be even larger than established polyploids. Our study showed that the changes in floral scent induced by polyploidization are more subtle than changes in floral morphology and to a lesser extent than established tetraploids differed from diploids. However, not only the scent emission rate and the number of scent compounds (analogous to floral size) were affected by polyploidization but also scent composition (analogous to floral shape). In *Heuchera grossulariifolia*– a close relative of *L. bolanderi*, phenotypic selection may reinforce divergence between polyploids and diploids (Nuismer and Cunningham 2005). Together with these findings, our results indicate that the immediate effects of polyploidization on floral scent seem to be less pronounced than for morphological traits and that reinforcement through subsequent evolutionary processes might be stronger for floral scent than for floral morphology.

As floral scent is an important pollinator attractant in many systems (Dötterl and Gershenzon 2023), including in *L. bolanderi* (Friberg et al. 2014), our finding that polyploidization can immediately induce changes in floral scent is key to understand the evolutionary ecology of plant-pollinator interactions in mixed-ploidy species. Future behavioral assays are needed to evaluate whether the differences between diploid and polyploid *L. bolanderi* are substantial enough to be detected by the specialized *Greya* moth pollinators. Thus far, two lines of evidence indicate such a potential. Firstly, an ecophysiological study identified 17 compounds in the floral scent of *L. bolanderi* that are physiologically active in *G. politella* moths (Schiestl et al. 2021), including compounds that differed in relative abundances among our ploidy-treatment groups (see Fig. S1). Secondly, behavioral assays show that different populations of *G. politella* and its congener *G. obscura* respond to differences in floral scent among local and non-local *Lithophragma* hosts (Friberg et al. 2014, 2016). Thus, there is a potential for pollinator-mediated selection on floral scent to reinforce the divergence in floral scent induced by polyploidization in newly emerged polyploid lines from their diploid progenitors and facilitate their establishment in *L. bolanderi*. Overall, our study shows that polyploidization may contribute to floral trait diversification by altering the number, composition, and emission rate of floral volatiles, all of which contribute to the diversification of interactions between plants and pollinators.

## Supplementary Information

Below is the link to the electronic supplementary material.

Supplementary Material 1 (PDF 957 KB)

## Data Availability

All datasets supporting the analysis and conclusions of the paper are available on Dryad at 10.5061/dryad.37pvmcvxx.
