# Supplementary material for: Direct Effects of Polyploidization on Floral Scent
Source: J Chem Ecol. 2025 Sep 10;51(5):89. doi: 10.1007/s10886-025-01641-y (PMC12423175; doi:10.1007/s10886-025-01641-y)
Supplement: Supplementary file 1 — Supplementary Material 1 (PDF 957 KB) [file 10886_2025_1641_MOESM1_ESM.pdf]

SUPPLEMENTARY INFORMATION

Direct Effects of Polyploidization on Floral Scent

Elisabeth Schlager[1], Stefan Dötterl[1], John N Thompson[2], Magne Friberg[3], Karin Gross[1,3,*]

[1] *Department of Environment and Biodiversity, University of Salzburg, Hellbrunner Strasse 34, 5020 Salzburg, Austria*

[2] *Department of Ecology and Evolutionary Biology, University of California, Santa Cruz, Santa Cruz, CA 95060, USA*

[3] *Department of Biology, Lund University, Kontaktvägen 13, SE-223 62 Lund, Sweden*

[*] *Corresponding author: karin.gross@plus.ac.at*

Text S1 – Sowing and plant growing conditions

The F0 plants were grown from seeds of plants of known ploidy level (Gross et al. 2025). They were grown in two cohorts – the first sown in February 2019 and the second sown in December 2019, because sample sizes of plants reaching the flowering stage were too low in the first cohort due to a high mortality, most likely caused by the colchicine treatment. In total, we sowed 2414 seeds from 9 diploid seed families from the population KAW with 174-300 seeds per seed family and 660 seeds from 11 tetraploid seed families from the population KAW with 60 seeds per seed family. Twenty seeds each per seed family were sown into a 9×9×7-cm plastic pots filled with S-jord (Hasselfors, Granngården, Sweden) potting soil. The newly sowed pots of the first cohort were kept in a small greenhouse room of the greenhouse facility of Lund University at a 14 h light/10 h dark rhythm (165 µmol m^{-2} s^{-1} light intensity during the light phase) and at approximately 15°C. The newly sowed pots of the second cohort were kept in growth chambers (Micro Clima-Series™ Economic Lux Chamber, Snijders Labs, The Netherlands) also at a 14 h light/10 h dark rhythm (165 µmol m^{-2} s^{-1} light intensity during the light phase; air humidity: 60%) and at 15°C. Pots were covered with transparent plastic foil to avoid drying out and watered approximately twice a week.

After the colchicine treatment, the seedlings were planted into individual wells (bottom ø: 2.8 cm; top ø: 4 cm; height: 4 cm) of 66-well trays filled with wetted S-jord and kept in the greenhouse (first cohort) and the growth chambers (second cohort). The conditions were as described above. When the seedlings were well established, we transplanted them into individual 9×9×7-cm plastic pots, that is, one seedling per pot. The pots had been filled with wetted S-jord potting soil, and a few nutrient balls had been mixed into the soil of each pot. The plants of the first cohort were kept in the same small greenhouse room under the conditions described and the plants of the second cohort were kept in the growth chambers under the conditions described until they were well established. Then, they were moved to a

larger greenhouse also at a 14 h light/10 h dark rhythm (natural and artificial light during light phase) at 15°C, and they were automatically watered every third day.

The F1 plants were also grown in two cohorts – the first cohort was sown in March 2021 and the second in December 2021 – because of a high mortality in the first cohort most likely caused by unexpected high temperatures in the greenhouse. In total, we sowed approximately 880 seeds from 60 crossings (43 donor plant × receiver plant combinations) for the colchicine-treated group, approximately 610 seeds from 33 crossings (29 donor plant × receiver plant combinations) for the diploid control group, and approximately 630 seeds from 32 crossings (32 donor plant × receiver plant combinations) for the tetraploid control group. In the first cohort, approximately 20 seeds per crossing of colchicine-treated plants, of diploid control plants, and of tetraploid control plants were sown into round plastic pots (9×7.5 cm) filled with wetted soil. We manually mixed the substrate of 15 parts soil (Einheitserde - BL Bio T Öko torffrei, Einheitserdewerke Werkverband e.V., Sinntal-Altengronau, Germany), one part sand (silica), and two parts perlite (PERLIGRAN ® Extra; Knauf Aquapanel GmbH, Dortmund, Germany) so that it was similar to S-jord. After approximately two months, we transplanted up to two seedlings per pot into individual plastic pots. In the second cohort, four seeds per crossing of colchicine-treated plants, of diploid control plants, and of tetraploid control plants were sown into individual pots (4×4×5 cm) of plastic seed trays consisting of 3×4 pots per tray. These pots had been filled with the same wetted substrate as in the first cohort except that another Einheitserde soil was used (Einheitserde CL ED73, Einheitserdewerke Werkverband e.V., Sinntal-Altengronau, Germany). Starting approximately two months after sowing, the most established plants were transferred into larger individual round plastic pots (9×7.5 cm), that is, one plant per pot. Newly sowed pots of both cohorts were kept in a climate chamber (Sanyo Growth Cabinet MLR-350T, Sanyo Electric Co. Ltd., Japan) at a 14 h light/10 h dark rhythm at 15°C during the light phase and at

10°C during the dark phase. The freshly transplanted plants of the first cohort were initially moved to a greenhouse of the greenhouse facility of the University of Salzburg, where natural light was augmented with artificial light for 14 h per day, the temperature was ≥ 15°C, and plants were spot-watered daily. The outdoor temperatures were exceptionally high for this time of year, and, as the plants did not do well under these conditions, they were moved back into climate chambers (one Sanyo Growth Cabinet MLR-350T, Sanyo Electric Co. Ltd., Japan; two Liebherr UKS 5000, Strauss, Salzburg, Austria) after six to eleven days. The temperature and light settings were as described above. The plants subsequently transplanted and the plants of the second cohort were kept in the climate chambers all the time.

Text S2 – Colchicine treatment

Approximately 4.5-7 weeks after sowing, we carefully picked diploid seedlings out of the soil in the pot using tweezers. We carefully swayed the roots of the seedlings in water to remove as much soil as possible in water and then carefully dabbed them on paper towel to remove as much water as possible. We then placed up to 30 seedlings of a seed family, depending on the size of the seedlings, into a plastic Petri dish (ø 5 cm). We completely submersed the seedlings in the Petri dishes in a 0.2% colchicine solution (2 mg colchicine powder [97%, Fisher Scientific, Art.-nr.: 10174953] in 1 ml deionized H_2O), which corresponded to approximately 5-10 ml depending on the size of the seedlings. The seedlings were incubated in the colchicine solution at room temperature in a fume hood darkened with black covering for approximately 16 h. Thereafter, we hold the seedlings with a pair of tweezers and swiveled them consecutively in three 100-ml glass bottles filled with deionized H_2O to rinse the colchicine solution. We then placed the rinsed seedlings into clean plastic Petri dishes (ø 5 cm). We treated a subset of the diploid and tetraploid seedling with deionized H_2O instead of the colchicine solution to generate the diploid and tetraploid control group, respectively. In

total, we treated 793 seedlings from 9 diploid seed families with 32-139 seedlings per seed family with the colchicine solution constituting the colchicine-treated plants, 87 seedlings from 9 diploid seed families with 8-10 seedlings per seed family with deionized H_2O constituting the diploid control plants, and 149 seedlings from 11 tetraploid seed families with 6-15 seedlings per seed family with deionized H_2O constituting the tetraploid control plants.

Text S3 – Scent collection and analysis

Flowers were enclosed in an oven bag (8 × 30 cm; Toppits® Frying Tube, Melitta, Germany) for dynamic headspace sorption. Air from the headspace was sucked through a scent trap (small glass tube [diameter: 1.9 mm] containing 3 mg of a 1:1 mixture of Tenax-TA [mesh 60-80; Supleco, Germany] and Carbotrap B [mesh 20-40; Suplec, Germany]) for 15 minutes with a rotary vane pump (G12/01; Gardner Denver Austria GmbH, Austria) at a constant flow rate of 200 ml min^{-1}, which was checked using an LPM Air flow meter (Brooks Instrument, Hatfield, PA, USA). Samples were stored in a freezer (-20°C) until gas chromatography/mass spectrometry (GC/MS) analysis.

The samples were analyzed using a GC/MS-QP2010 Ultra (Shimadzu, Japan) equipped with a TD-20 (Shimadzu, Japan) thermal desorption autosampler and a ZB-5 fused silica column (Zebron™, Phenomenex Inc., USA; 5% phenyl, 95% dimethylpolysiloxane; 0.25 mm diameter, 0.25 µm film thickness, 60 m length). Helium served as carrier gas at a constant velocity of 1.5 ml min^{-1}, and the split ratio was 1:1 with two exceptions (1:3 and 1:5), which were adjusted for further analysis using comparative series with linalool. The start temperature was 40°C in the GC oven, the temperature increase was 6°C min^{-1} to 250°C, and the end temperature was held for 1 min. The MS interface was operated at 260°C and the ion source at 200°C. Mass-to-charge ratios (m/z) were taken from 34 to 350 at 70 eV electron ionization (EI) mass spectra.

References

Adams RP (2007) Identification of essential oil components by gas chromatography/quadrupole mass spectroscopy, 4th edition. Allured Publishing Corporation, Carol Stream, IL, USA

Gross K, Yazdi HP, Schlager E, et al (2025) Repeated polyploidization shapes divergence in floral morphology in *Lithophragma bolanderi* (Saxifragaceae). Proceedings of the National Academy of Sciences 122:e2505119122.

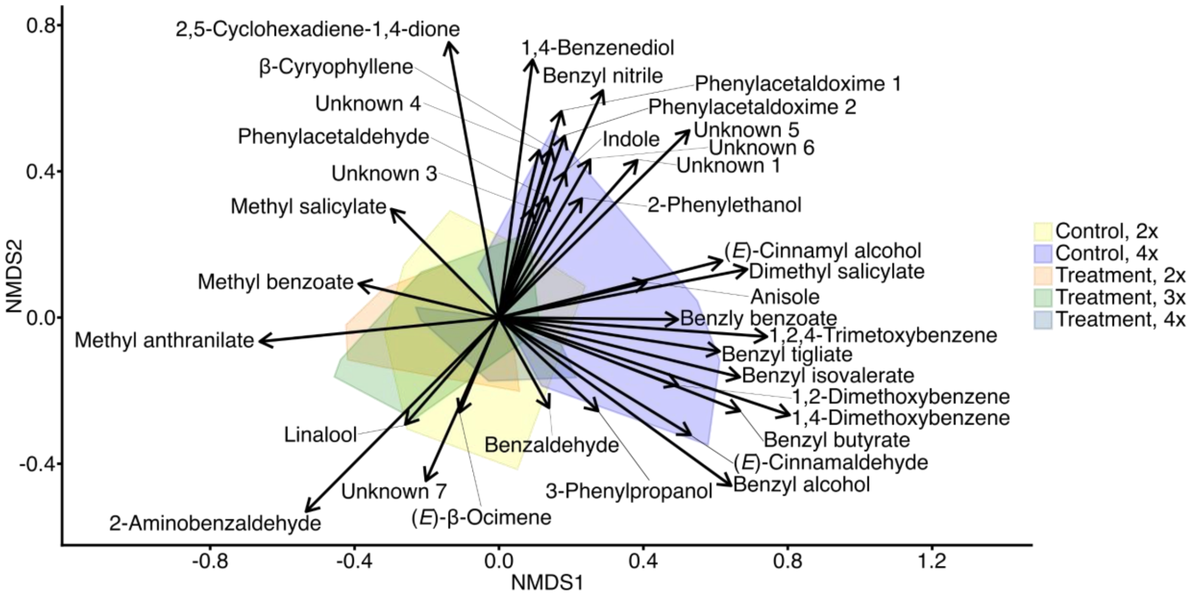

Fig. S1 Non-metric multidimensional scaling (NMDS) scores for NMDS1 and NMDS2 of the floral scent compounds of *Lithophragma bolanderi*. Only compounds with significant correlations with NMDS1 and NMDS2 are indicated. For the correlations of all compounds, see Table S2. Perimeters of the established diploids ("Control, 2x") and established tetraploids ("Control, 4x") as well as of neopolyploids generated through colchicine treatment of diploid seedlings ("Treatment, 3x": neotriploids, and "Treatment, 4x": neotetraploids) and colchicine-treated plants that remained diploid ("Treatment, 2x") are also indicated as a guidance and are the same as in Fig. 1.

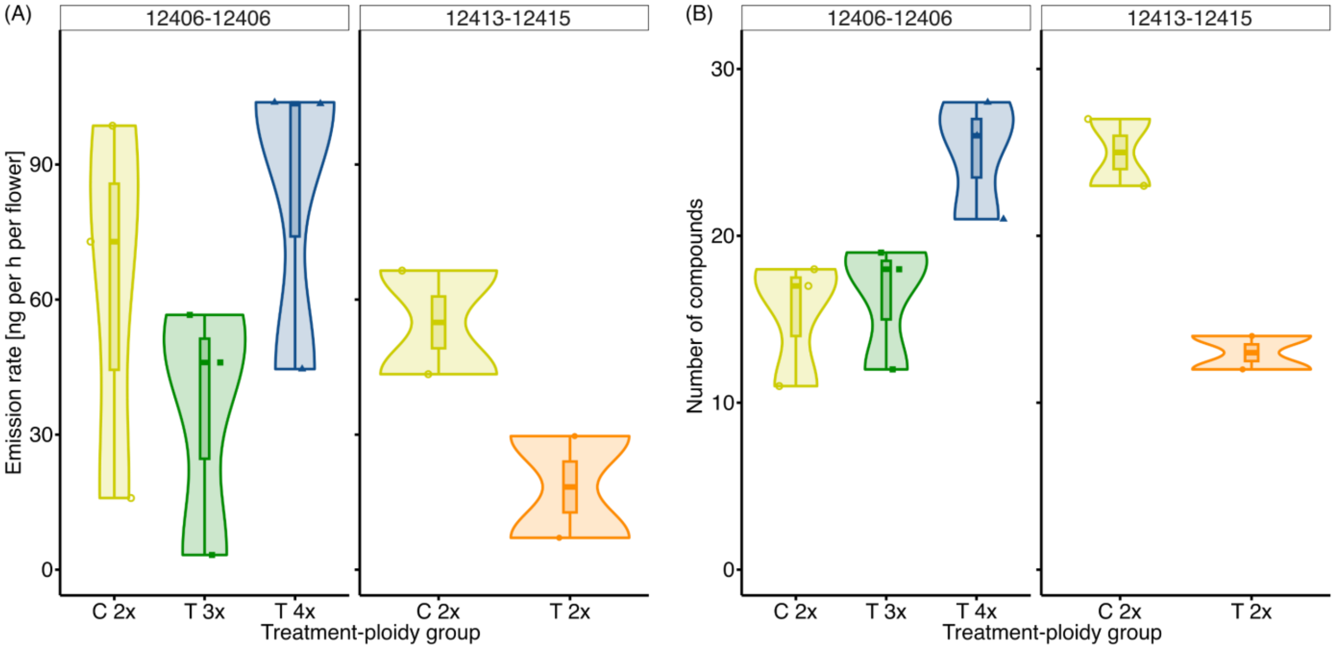

Fig. S2 Cross-specific differentiation in scent emission rate (**A**) and number of floral scent compounds (**B**) of *Lithophragma bolanderi* among cytotype-treatment groups. For the cross 12406-12406 (ID of the donor seed family – ID of the receiver seed family; left), there were three established diploids (C 2x), three neotetraploids (T 4x), and three neotriploids (T 3x), and, for the cross 12413-12415 (right), there were two established diploids (C 2x) and two colchicine-treated plants that remained diploid (T 2x). Points representing individuals, violin plots, and boxplots are color-coded according to treatment and cytotype. Boxplots indicate the median, the first and third quartile, maximum and minimum values. The statistics of the global tests are listed in Table S4, and all pairwise post-hoc comparisons were non-significant (PERMANOVA: $P > 0.05$). Neither dispersion in total absolute amount of scent (**A**; PERDISP: $F_{2,6} = 1.71$, $P = 0.664$) nor dispersion in the number of scent compounds (**B**; PERDISP: $F_{2,6} = 0.75$, $P = 0.728$) differ among cytotypes (left).

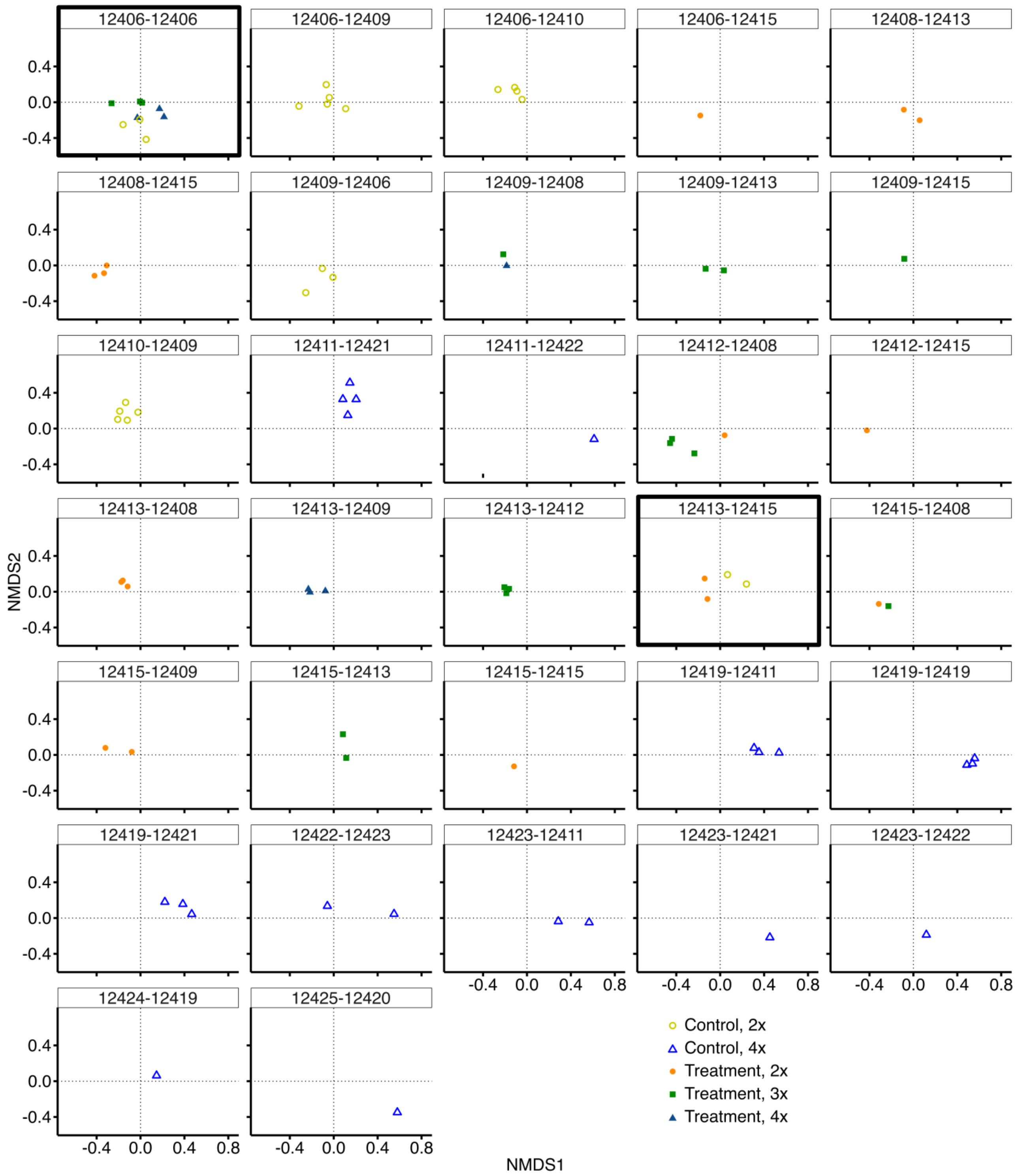

Fig. S3 Differentiation in relative amounts of floral scent compounds of *Lithophragma bolanderi* among cytotypes (2x: diploid, 3x: triploid, 4x: tetraploid) and between colchicine-treated (Treatment) and untreated (Control) groups according to non-metric multidimensional scaling (NMDS) axis 1 and 2, and separately for each donor-receiver seed family combination (donor seed family-receiver seed family). Symbols represent individuals and are color-coded according to cytotype and shaped according to treatment. Floral scent compound loadings on NMDS1 and NMDS2 are shown in Fig. S1 and Table S2. Cross-specific seed families with at least two of the five groups with at least two individuals per group (framed with a thick line) were used for cross-specific analyses to control for founder effects. All pairwise post-hoc comparisons were non-significant (PERMANOVA: $P > 0.05$). Multivariate dispersion in relative amounts did not differ among cytotypes in the crossing 12406-12406 (PERDISP: $F_{2,6} = 5.03$, $P = 0.175$).

Table S1 Overall occurrence of floral scent compounds and the occurrence and median (minimum-maximum) relative amount [%] of floral scent compounds separately for established diploids (Control, 2x) and tetraploids (Control, 4x), colchicine-treated plants that remained diploid (Treatment, 2x), neotriploids (Treatment, 3x), and neotetraploids (Treatment, 4x) of *Lithophragma bolanderi*. Compounds are ordered according to chemical compound class and within class according to the linear retention index (RI). Sample sizes are also given. The identification method indicates whether they were identified with authentic reference compounds available in the Plant Ecology Lab at the University of Salzburg (Yes) or through library suggestions based on published RIs and mass spectra in the data bases NIST11, Wiley9, Essential oils, FFNSC 2, and Adams (2007) (No). For unknown compounds, the most common mass spectral ion fragments are given including the relative contribution in % in parentheses.

			Control				Treatment					
			2x (*n* = 22)		4x (*n* = 22)		2x (*n* = 17)		3x (*n* = 16)		4x (*n* = 7)	
Compound class and name	Authentic standard available	RI	Occurrence	Median (min-max) relative amount [%]	Occurrence	Median (min-max) relative amount [%]	Occurrence	Median (min-max) relative amount [%]	Occurrence	Median (min-max) relative amount [%]	Occurrence	Median (min-max) relative amount [%]
FATTY ACID DERIVATIVES												
2,4-Hexadiene	Yes	646	14	0.031 (0-0.341)	2	0 (0-0.051)	8	0 (0-0.287)	8	0.001 (0-0.815)	6	0.109 (0-0.578)
(Z)-3-Hexen-1-ol	Yes	854	10	0 (0-2.316)	10	0 (0-4.091)	5	0 (0-0.992)	10	0.317 (0-1.415)	5	0.140 (0-0.519)
(E)-4-Oxohex-2-enal	No	953	6	0 (0-2.293)	6	0 (0-0.088)	3	0 (0-0.211)	4	0 (0-0.172)	5	0.019 (0-0.053)
BENZENOIDS AND PHENYL PROPANOIDS												
Benzaldehyde	Yes	963	5	0 (0-8.714)	2	0 (0-8.717)	0	0 (0-0)	0	0 (0-0)	3	0 (0-17.583)
Benzyl alcohol	Yes	1035	7	0 (0-5.896)	19	2.590 (0-10.746)	4	0 (0-4.813)	4	0 (0-4.935)	2	0 (0-6.002)
Phenylacetaldehyde	Yes	1048	1	0 (0-0.006)	3	0 (0-0.176)	0	0 (0-0)	0	0 (0-0)	0	0 (0-0)
2-Methoxyphenol	Yes	1094	7	0 (0-0.190)	4	0 (0-0.0767)	1	0 (0-0.013)	2	0 (0-0.017)	2	0 (0-0.006)
Methyl benzoate	Yes	1101	22	1.738 (0.665-7.132)	22	0.998 (0.080-2.621)	17	1.596 (0.369-3.157)	16	1.810 (0.706-6.442)	7	2.248 (1.205-4.896)
2-Phenylethanol	Yes	1118	0	0 (0-0)	6	0 (0-0.198)	0	0 (0-0)	0	0 (0-0)	0	0 (0-0)
1,2-Dimethoxybenzene	Yes	1146	4	0 (0-2.327)	12	0.030 (0-0.363)	1	0 (0-0.206)	1	0 (0-0.366)	0	0 (0-0)
1,4-Dimethoxybenzene	Yes	1167	1	0 (0-2.358)	18	12.865 (0-73.706)	0	0 (0-0)	0	0 (0-0)	0	0 (0-0)
Methyl salicylate	Yes	1202	22	19.331 (6.219-41.149)	22	11.384 (2.466-45.165)	17	19.480 (8.350-49.112)	16	22.460 (9.174-48.255)	7	21.071 (12.60-33.362)
3-Phenylpropanol	Yes	1235	3	0 (0-0.640)	7	0 (0-0.361)	1	0 (0-0.417)	2	0 (0-1.308)	3	0 (0-0.531)
1,4-Benzenediol	Yes	1264	19	1.005 (0-4.074)	18	0.836 (0-2.873)	8	0 (0-4.166)	10	0.334 (0-2.155)	7	0.555 (0.031-1.590)
(E)-Cinnamaldehyde	Yes	1279	9	0 (0-5.850)	18	0.535 (0-4.408)	5	0 (0-3.478)	4	0 (0-12.238)	4	0.159 (0-3.941)
Anisole	No	1288	0	0 (0-0)	4	0 (0-0.105)	0	0 (0-0)	0	0 (0-0)	0	0 (0-0)
(E)-Cinnamyl alcohol	Yes	1313	0	0 (0-0)	14	0.169 (0-7.768)	1	0 (0-0.261)	1	0 (0-0.590)	2	0 (0-0.220)
Dimethyl salicylate	Yes	1343	10	0 (0-3.755)	20	0.339 (0-1.837)	5	0 (0-0.364)	8	0.023 (0-7.936)	6	0.047 (0-0.1379)
Benzyl butyrate	Yes	1351	1	0 (0-0.009)	13	0.020 (0-0.660)	1	0 (0-0.018)	0	0 (0-0)	1	0 (0-0.0427)
1,2,4-Trimethoxybenzene	Yes	1374	4	0 (0-0.173)	15	0.312 (0-4.267)	1	0 (0-0.292)	1	0 (0-0.264)	1	0 (0-0.159)
Benzyl isovalerate	No	1402	14	0.006 (0-0.159)	20	0.069 (0-1.013)	7	0 (0-0.074)	8	0.001 (0-0.126)	4	0.008 (0-0.093)
Benzyl tiglate	Yes	1508	3	0 (0-0.072)	13	0.017 (0-0.170)	3	0 (0-0.063)	3	0 (0-0.197)	2	0 (0-0.042)
Benzyl benzoate	Yes	1783	15	0.065 (0-0.714)	17	0.102 (0-0.978)	7	0 (0-0.238)	10	0.059 (0-0.265)	5	0.153 (0-0.328)
MONOTERPENES												
(*E*)-β-Ocimene	Yes	1050	3	0 (0-0.075)	0	0 (0-0)	3	0 (0-0.209)	3	0 (0-0.076)	1	0 (0-0.130)
Linalool	Yes	1101	22	0.690 (0.067-4.563)	22	0.406 (0.065-5.168)	17	2.344 (0.092-10.053)	16	2.359 (0.415-14.320)	7	1.205 (0.598-10.181)
Hotrienol	No	1106	4	0 (0-0.545)	0	0 (0-0)	1	0 (0-0.075)	6	0 (0-0.272)	4	0.019 (0-0.359)
SESQUITERPENES												
β-Caryophyllene	Yes	1437	1	0 (0-0.156)	10	0 (0-1.681)	2	0 (0-0.131)	1	0 (0-0.146)	2	0 (0-0.310)
C5-BRANCHED CHAIN COMPOUNDS												
Isoamyl alcohol	Yes	730	0	0 (0-0)	2	0 (0-0.507)	1	0 (0-3.207)	0	0 (0-0)	1	0 (0-0.249)
NITROGEN CONTAINING COMPOUNDS												
Benzyl nitrile	Yes	1138	11	0.016 (0-0.674)	20	0.133 (0-3.466)	5	0 (0-1.010)	6	0 (0-0.314)	3	0 (0-0.059)
2-Aminobenzaldehyde	Yes	1234	22	28.896 (1.435-69.849)	22	12.242 (0.371-51.837)	17	35.400 (20.311-66.352)	16	36.402 (10.424-66.796)	7	37.187 (23.476-45.007)
Phenylacetaldoxime 1	Yes	1256	5	0 (0-0.194)	8	0 (0-1.021)	0	0 (0-0)	0	0 (0-0)	0	0 (0-0)
Phenylacetaldoxime 2	Yes	1278	4	0 (0-0.1613996)	7	0 (0-0.811)	0	0 (0-0)	0	0 (0-0)	0	0 (0-0)
Indole	Yes	1305	22	2.013 (0.213-13.188)	22	2.203 (0.172-20.565)	16	1.285 (0-5.262)	14	1.444 (0-4.340)	7	1.499 (1.215-3.658)
Methyl anthranilate	Yes	1352	22	8.017 (1.298-21.851)	22	2.369 (0.029-16.179)	17	10.293 (3.140-19.958)	16	8.781 (3.172-32.541)	7	13.840 (2.721-23.672)
MICELLANEOUS CYCLIC COMPOUNDS												
2,5-Cyclohexadiene-1,4-dione	Yes	920	21	27.650 (0-46.215)	22	14.831 (1.611-59.227)	17	22.092 (7.977-41.897)	16	12.556 (3.452-27.998)	7	15.792 (9.602-21.370)
UNKNOWN												
Unknown 1<br>[43(55%),57(24%),71(100%),88(16%)]	No	1018	0	0 (0-0)	15	0.037 (0-0.779)	0	0 (0-0)	0	0 (0-0)	1	0 (0-0.002)
Unknown 2<br>[41(35%),43(57%),67(64%),68(100%),<br>83(53%),85(63%)]	No	1262	9	0 (0-1.327)	0	0 (0-0)	5	0 (0-0.271)	4	0 (0-0.459)	4	0.182 (0-1.250)
Unknown 3<br>[55(70%),68(75%),69(68%),81(47%),82(57%),<br>95(45%),124(100%)]	No	1342	22	0.256 (0.059-1.044)	22	0.185 (0.049-2.807)	15	0.258 (0-0.796)	14	0.237 (0-1.214)	7	0.171 (0.093-0.904)
Unknown 4<br>[41(57%),55(76%),68(56%),69(63%),<br>82(44%),124(100%),166(18%),193(14%)]	No	1379	18	0.059 (0-0.397)	19	0.028 (0-0.344)	9	0.027 (0-0.828)	12	0.060 (0-1.012)	4	0.021 (0-0.147)
Unknown 5<br>[107(28%),150(100%),182(33%)]	No	1451	2	0 (0-0.132)	20	0.047 (0-0.831)	0	0 (0-0)	1	0 (0-0.177)	0	0 (0-0)
Unknown 6<br>[119(24%),161(40%),179(100%)]	No	1633	0	0 (0-0)	9	0 (0-0.277)	0	0 (0-0)	0	0 (0-0)	0	0 (0-0)
Unknown 7<br>[57(100%),93(19%),121(16%),147(42%)]	No	1705	2	0 (0-0.050)	2	0 (0-0.031)	3	0 (0-0.344)	4	0 (0-0.360)	1	0 (0-0.008)

Table S2 Non-metric multidimensional scaling (NMDS) scores of the floral scent compounds of *Lithophragma bolanderi* for NMDS1 and NMDS2 as well as the unite length vectors, their correlation (square root of r^2), and their significance value (*P*). Compounds are ordered as in Table S2.

	Scores		Unite length vector			
Compound class and name	NMDS1	NMDS2	NMDS1	NMDS2	r^2	*P*
FATTY ACID DERIVATIVES						
2,4-Hexadiene	-0.190	-0.025	-0.994	-0.114	0.037	0.219
(Z)-3-Hexen-1-ol	0.089	-0.096	0.731	-0.682	0.068	0.059
(E)-4-Oxohex-2-enal	0.187	-0.095	0.878	-0.478	0.063	0.075
BENZENOIDS AND PHENYL PROPANOIDS						
Benzaldehyde	0.260	-0.350	0.490	-0.872	0.080	**0.036**
Benzyl alcohol	0.453	-0.232	0.813	-0.582	0.625	**<0.001**
Phenylacetaldehyde	0.495	0.789	0.376	0.926	0.126	**0.006**
2-Methoxyphenol	0.106	0.281	0.308	0.951	0.070	0.057
Methyl benzoate	-0.099	-0.010	-0.973	0.231	0.160	**<0.001**
2-Phenylethanol	0.607	0.543	0.576	0.818	0.159	**0.002**
1,2-Dimethoxybenzene	0.548	-0.150	0.937	-0.349	0.280	**<0.001**
1,4-Dimethoxybenzene	0.946	-0.213	0.949	-0.314	0.719	**<0.001**
Methyl salicylate	-0.089	0.003	-0.709	0.705	0.177	**<0.001**
3-Phenylpropanol	0.349	-0.240	0.732	-0.682	0.141	**0.002**
1,4-Benzenediol	0.003	0.253	0.131	0.991	0.506	**<0.001**
(E)-Cinnamaldehyde	0.339	-0.158	0.856	-0.518	0.385	**<0.001**
Anisole	1.029	0.136	0.973	0.230	0.176	**0.001**
(E)-Cinnamyl alcohol	0.700	0.097	0.970	0.244	0.406	**<0.001**
Dimethyl salicylate	0.342	0.029	0.982	0.189	0.490	**<0.001**
Benzyl butyrate	0.838	-0.219	0.934	-0.358	0.511	**<0.001**
1,2,4-Trimethoxybenzene	0.703	-0.048	0.998	-0.070	0.554	**<0.001**
Benzyl isovalerate	0.291	-0.066	0.971	-0.237	0.472	**<0.001**
Benzyl tiglate	0.530	-0.069	0.989	-0.150	0.382	**<0.001**
Benzyl benzoate	0.173	-0.018	1.000	-0.011	0.245	**<0.001**
MONOTERPENES						
trans-β-Ocimene	-0.318	-0.384	-0.388	-0.921	0.079	**0.035**
Linalool	-0.116	-0.056	-0.664	-0.748	0.153	**0.001**
Hotrienol	-0.260	-0.125	-0.743	-0.669	0.031	0.274
SESQUITERPENES						
β-Caryophyllene	0.229	0.522	0.300	0.954	0.234	**<0.001**
C5-BRANCHED CHAIN COMPOUNDS						
Isoamyl alcohol	0.176	-0.620	0.223	-0.975	0.056	0.088
NITROGEN CONTAINING COMPOUNDS						
Benzyl nitrile	0.195	0.307	0.420	0.908	0.469	**<0.001**
2-Aminobenzaldehyde	-0.129	-0.059	-0.710	-0.705	0.568	**<0.001**
Phenylacetaldoxime 1	0.329	0.730	0.293	0.956	0.348	**<0.001**
Phenylacetaldoxime 2	0.377	0.688	0.343	0.939	0.280	**<0.001**
Indole	-0.010	0.043	0.419	0.908	0.192	**<0.001**
Methyl anthranilate	-0.146	-0.021	-0.995	-0.099	0.444	**<0.001**
MICELLANEOUS CYCLIC COMPOUNDS						
2,5-Cyclohexadiene-1,4-dione	-0.086	0.079	-0.182	0.983	0.586	**<0.001**
UNKNOWN						
Unknown 1	0.553	0.390	0.665	0.747	0.332	**<0.001**
Unknown 2	-0.199	0.060	-0.743	0.669	0.025	0.352
Unknown 3	-0.030	0.035	0.291	0.957	0.096	**0.017**
Unknown 4	0.014	0.156	0.236	0.972	0.222	**<0.001**
Unknown 5	0.585	0.354	0.717	0.697	0.540	**<0.001**
Unknown 6	0.567	0.617	0.504	0.864	0.250	**<0.001**
Unknown 7	-0.503	-0.601	-0.413	-0.911	0.241	**<0.001**

Table S3 Statistical output of the pairwise *posthoc* comparisons of permutational multivariate analyses of variance (PERMANOVAs) including uncorrected and Benjamin-Hochberg corrected *P*-values. Significant *P*-values are highlighted in bold. “control_2x” established diploids, “control_4x” established tetraploids, “treatment_2x” colchicine-treated plants that remained diploid, “treatment_3x” neotriploids, and “treatment_4x” neotetraploids.

				P-value	
Trait	Pairwise comparison	*t*	No. unique permutations	Uncorrected	Benjamini-Hochberg corrected
Scent emission rate					
	control_2x - treatment_3x	1.20	9843	0.231	0.329
	control_2x - treatment_2x	1.89	9809	0.072	0.143
	control_2x - control_4x	2.99	9849	**0.007**	**0.034**
	control_2x - treatment_4x	1.04	9832	0.314	0.349
	treatment_3x - treatment_2x	0.62	9832	0.536	0.536
	treatment_3x - control_4x	2.12	9830	**0.042**	0.106
	treatment_3x - treatment_4x	1.50	9855	0.154	0.256
	treatment_2x - control_4x	3.42	9821	**0.002**	**0.015**
	treatment_2x - treatment_4x	2.54	9840	**0.033**	0.106
	control_4x - treatment_4x	1.09	9853	0.296	0.349
Number of compounds					
	control_2x - treatment_3x	0.41	9858	0.686	0.686
	control_2x - treatment_2x	3.65	9808	**0.001**	**0.003**
	control_2x - control_4x	3.98	9833	**0.001**	**0.003**
	control_2x - treatment_4x	3.42	9840	**0.003**	**0.006**
	treatment_3x - treatment_2x	2.60	9820	**0.022**	**0.031**
	treatment_3x - control_4x	5.21	9863	**<0.001**	**0.001**
	treatment_3x - treatment_4x	2.73	9849	**0.017**	**0.029**
	treatment_2x - control_4x	6.08	9835	**<0.001**	**0.001**
	treatment_2x - treatment_4x	1.80	9784	0.102	0.113
	control_4x - treatment_4x	2.14	9819	**0.042**	0.053
Relative amounts					
	control_2x - treatment_3x	1.42	9946	0.064	0.071
	control_2x - treatment_2x	1.57	9947	**0.041**	0.051
	control_2x - control_4x	3.07	9954	**<0.001**	**0.001**
	control_2x - treatment_4x	1.63	9952	**0.026**	**0.040**
	treatment_3x - treatment_2x	1.95	9958	**0.012**	**0.024**
	treatment_3x - control_4x	3.36	9945	**<0.001**	**0.001**
	treatment_3x - treatment_4x	1.66	9950	**0.028**	**0.040**
	treatment_2x - control_4x	3.82	9940	**<0.001**	**0.001**
	treatment_2x - treatment_4x	1.14	9915	0.239	0.239
	control_4x - treatment_4x	2.31	9946	**0.008**	**0.020**

Table S4 Mean ± SE (standard error) dispersion and statistical output of permutational analyses of multivariate dispersion (PERMDISPs) for the five cytotype-treatment groups – established diploids (Control, 2x), established tetraploids (Control, 4x), colchicine-treated plants that remained diploid (Treatment, 2x), neotriploids (Treatment, 3x), and neotetraploids (Treatment, 4x) – in *Lithophragma bolanderi*. Significant *P*-values are highlighted in bold. Different lowercase letters indicate significant ($P < 0.05$) differences in pairwise comparisons of cytotypes.

	Mean ± SE dispersion					Statistics	
	Control		Treatment				
Trait	2x	4x	2x	3x	4x	$F_{4,79}$	P
Scent emission rate							
	0.27 ± 0.05 [a]	0.55 ± 0.08 [b]	0.32 ± 0.06 [a]	0.43 ± 0.10 [a,b]	0.25 ± 0.04 [a]	2.97	**0.032**
Number of compounds							
	0.08 ± 0.02 [a,b]	0.07 ± 0.01 [a]	0.13 ± 0.02 [b]	0.11 ± 0.02 [a,b]	0.14 ± 0.03 [b]	2.32	0.077
Relative amounts							
	18.87 ± 1.12 [a,b]	20.59 ± 1.10 [a]	19.91 ± 0.81 [b]	17.90 ± 1.16 [a,b]	16.84 ± 1.55 [a,b]	1.9	0.174

Table S5 Statistical output of permutational multivariate analyses of variance (PERMANOVAs) including cytotype (Ploidy; diploid, neotriploid, neotetraploid) for the donor-receiver seed family combination (Crossing) 12406-12406 and treatment (Treatment; colchicine-treated plants that remained diploid, established diploids) for the donor-receiver seed family combination 12413-12415 as fixed factor. The proportion of the total variance explained (R^2 [%]) is also indicated. Significant *P*-values are highlighted in bold.

	Crossing 12406 - 12406								Crossing 12413 - 12415							
Trait	Factor	df	SS	MS	Pseudo-*F*	*P*	No. unique permutations	R^2 [%]	Factor	df	SS	MS	Pseudo-*F*	*P*	No. unique permutations	R^2 [%]
Scent emission rate																
	Ploidy	2	0.87	0.44	1.20	0.377	280	28.6	Treatment	1	0.53	0.53	3.68	0.346	3	64.8
	Residuals	6	2.18	0.36				71.4	Residuals	2	0.29	0.14				35.2
	Total	8	3.06						Total	3	0.82					
Number of compounds																
	Ploidy	2	0.123	0.061	4.72	0.058	175	61.2	Treatment	1	0.114	0.114	33.97	0.338	3	94.4
	Residuals	6	0.078	0.013				38.8	Residuals	2	0.007	0.003				5.6
	Total	8	0.201						Total	3	0.120					
Relative amount																
	Ploidy	2	1262.5	631.2	1.88	**0.025**	280	38.6	Treatment	1	658.8	658.8	2.74	0.336	3	57.9
	Residuals	6	2011.1	335.2				61.4	Residuals	2	480.0	240.0				42.2
	Total	8	3273.5						Total	3	1138.8					
